# The Need for Cervical Cancer Control in HIV-Positive and HIV-Negative Women from Romania by Primary Prevention and by Early Detection Using Clinically Validated HPV/DNA Tests

**DOI:** 10.1371/journal.pone.0132271

**Published:** 2015-07-17

**Authors:** Ramona Gabriela Ursu, Mircea Onofriescu, Alexandru Luca, Liviu Jany Prisecariu, Silvia Olivia Sălceanu, Dragoş Nemescu, Luminiţa Smaranda Iancu

**Affiliations:** 1 Department of Microbiology, University of Medicine and Pharmacy “Gr. T. Popa”, Iaşi, Romania; 2 Department of Obstetrics and Gynecology, University of Medicine and Pharmacy “Gr.T.Popa”, Iasi, Romania, “Cuza Voda” Obstetrics and Gynecology Hospital, Iasi, Romania; 3 “Cuza Voda” Obstetrics and Gynecology Hospital, Iasi, Romania; 4 Infectious Disease “St. Parascheva” Clinical University Hospital Iaşi, Iaşi, Romania; 5 Clinical Hospital "Căi Ferate" Iaşi, Iaşi, Romania; Ruđer Bošković Institute, CROATIA

## Abstract

**Background:**

In Romania, a country with no organized national surveillance program regarding cervical cancer, the early diagnosis of HPV (Human Papilloma Virus) infections is a major requirement, especially in HIV-infected women. The objective of this study was to determine the HPV prevalence and type distribution in young HIV-positive women and to assess the difference in the risk factors for developing cervical cancer compared to those of HIV-negative women.

**Method:**

We conducted one cross-sectional cohort study from June 2013–September 2014, including 1,032 women: 992 HIV- women who were 36.5 years old (limits: 17 ÷ 84) and 40 HIV + women who were 22.9 years old (limits: 17 ÷ 30) with iatrogenic HIV infected. We detected HPV types with the *Linear Array HPV Genotyping* test (Roche, Romania).

**Results:**

DNA/HPV was detected in 18/40 (45%) of the HIV+ patients and in 350/992 (35.2%) of the HIV- patients (OR = 1.5, 95%CI 0.76÷2.96). After age adjustment, the overall HPV prevalence was 51.6% in HIV+ versus 63.2% in HIV- women aged under 25, and 22.2% in HPV+ versus 47.2% in HIV- women aged 25–34. We detect HIV being a risk factor for acquiring multiple HPV type infections (OR = 2.30, 95% CI 0.88÷5.97). The eight most common HPV types (high-risk, and low-risk) for women below age 30, HIV+ / - were: HPV 16, 18, 31, 51, 58, 68, and 6 and 82 respectively. To assess the risk factors of HIV-positive women for acquiring HPV infection, we analyzed the CD4/μL, ARN/HIV copies/μL, the age group, the number of sexual partners, smoking, and the type of HPV infection (single versus multiple infections). We found that the number of sexual partners and smoking are statistically significant risk factors.

**Conclusion:**

Even though there are no significant differences regarding the prevalence of HPV infection in HIV + *versus* HIV – patients, multiple infections were more frequent in the first group. In our study group young HIV-infected patients under HAART therapy, high number of sexual partners (more than 3) and smoking were detected to be risk factors. Future organized screening for HPV infection using sensitive and specific methods are necessary at the national level in Romania.

## Introduction

The primary cause of cervical cancer is persistent infection with high-risk human papillomavirus (HR HPV) types [1]. HPV is a common virus that is sexually transmitted, and most cervical HPV infections resolve spontaneously within 2 years. Cervical cancer is one of the main causes of cancer incidence and death in women, most notably in low- and middle-income countries [2]. According to GLOBOCAN 2012, Romania is the leading country in Europe regarding the incidence (28.6/100.000) of cervical cancer [3]. Special populations, like immunosuppressed individuals, pregnant women, and homosexuals are high risk groups for acquiring HPV infections [4].The therapy of HPV-related diseases in case of HIV-positive persons can be difficult and HIV status accelerates evolution to HPV-associated cancers [5].

In 1989, in Romania it was an important nosocomial HIV epidemic, when many institutionalized children contracted HIV through blood transfusions and/or infected needles [6]. On30 June 2014, the total number of HIV/AIDS cases (cumulative for the periodfrom1985-2014) in Romania was 19,696 [7].The ECDC 2013 Report reveals that the percentage of HIV diagnoses in young people was the highest for Romania, reporting that > 35% of the HIV diagnoses are among young people [8].

In Romania 2012, the national government attempted to implement organized and free cervical screening by cytology but there was no centralized quality control or specific training and audits. Even under these conditions, it was possible to detect approximately 120 new cases of cervical cancer annually (data not published). The program has been stopped because of the lack of financial and political support, and screening by Pap test is now possible by request. The HPV vaccination program also failed, even though *Cervical Cancer Action* states that in Romania, there is a national program for HPV vaccination [9].

In settings where HIV is endemic, screening for cervical cancer is particularly important. Although Romania is not included in the endemic area, HIV-positive patients represent a high-risk group for many infections, including HPV. The aim of this study was to evaluate the HPV type prevalence and distribution in HIV-positive women and to assess the difference in the risk factors for developing cervical cancer compared to those of HIV-negative women.

## Material and Methods

### Study population

We conducted one cross-sectional cohort study from June 2013- September 2014, including 1032HIV-negative women from “Cuza-Vodă” Gynecology Clinical University Hospital Iaşi and 40 HIV-positive/AIDS women from Infectious Disease “St. Parascheva” Clinical University Hospital Iaşi. The average age for 992HIV- women was 36.5 years old (limits: 17 ÷ 84) and 22.8 year old (limits: 17 ÷ 30) for40 HIV +patients. Half of the first study group (514 HIV-negative women respectively)was included in a previous study that was published in 2011 [10].The HIV-positive women were iatrogenically infected, and the medium period of treatment for the last therapy regimen was 5 years. The HAART therapy used the classic regim with 2–4 drugs: an association between two inhibitors of protease, nucleosidic inhibitors of reverse transcriptase, protease inhibitor and non-nucleosidic inhibitor of reverse transcriptase (kaletra, abacavir + lamivudine, invirase, zidovudine + lamivudine, ritonavir, didanosine, darunavir, andetravirine). All of the patients were monitored by clinical gynecological, cytology and colposcopic examinations. All HIV positive women were sexually active, and majority of those from HIV negative group (97.3%, bellow 70 years old, according with their answers from questionnaires); we also included in our study pregnant women (13 / 32.5% HIV-positive women and 16 / 1.6% HIV-negative women).

### Ethics statement

Thestudy was approved by the Bioethical Research Committee of the “Gr. T. Popa” University of Medicine and Pharmacy Iaşi. All participants in the study signed informed consent form after they were shown/presented the benefits of inclusion in this study and filled out a questionnaire that was administered by an interviewer.

### Data collection and laboratory procedures

The study participants completed a questionnaire concerning the known cofactors for cervical cancer (e.g., smoking, genital co-infections, oral contraceptive use, and number of sex partners) and provided cytological results of previous Pap tests. HPV genotyping was performed in the Virology Laboratory of “Gr. T. Popa” University of Medicine and Pharmacy, Iași, using the Linear Array (LA)HPV genotyping method as previously described (18). The LA method identified 37 HPV types: 13 high risk (HR) (16, 18, 31, 33, 35, 39, 45, 51, 52, 56, 58, 59, and 68)and 24 low or intermediate risk (LR) (6, 11, 26, 39, 40, 42, 45, 53, 54, 55, 61, 62, 64, 66, 67, 69, 70, 71, 72, 73, 81, 82, 83, 84, IS39, and CP6108).

### Statistical analysis

The age was categorized into the following groups: <25; 25–34; 35–44; 45–54; and 55+.years. Descriptive statistics were prepared for demographic variables: age, births, pregnancies, abortions, DNA/HPV presence, multiple HPV infections, and HR and LR HPV types. According to the presence or absence of HIV infection, after age adjustment, we used chi-2 test for OR, CI 95%, to determine statistically significant differences between the proportions for each variable. If OR was > 1, we considered the tested variable being a risk factors for acquiring HPV infections. The CD4+ cell count and ARN/HIV viral load were both divided into three groups (CD4/μL: < 200, 200–499, > 500), respectively ARN/HIV copies/mL: < 10,000, 10,000–1,000,000, >100,000), based on the median value for each respective variable. To assess the risk factors for HIV-positive women for developing cervical cancer, we used the Kruskal-Wallis test. Statistical analysis was performed using SPSS version 20.0 software. A *p* value of <0.05 was considered statistically significant.

## Results

For HIV-positive women, we detected 18/40 (45%) positive for DNA/HPV; the HPV prevalence for HIV-negative women was 35.2% (350/992).Among the HIV+ women, the most prevalent HR HPV were HPV 52 (12.5%), HPV 31 and 68 (each 10%), HPV 16 and 51 (7.5%) and HPV 18 (5%). For HIV-negative women, the descending order of HR HPV types was HPV 16 (11%), HPV 52 (4%), HPV 18 (3.9%), HPV 31 and 51 (3.7% each), and HPV 58 (2.75%). (S1 Table). There was a difference regarding the LR HPV types that were included in the vaccine formula: 10% HPV 6 and5% HPV 11 in HIV-positive women *versus* 2.3% HPV 6 and 0.19% HPV 11 in HIV-negative women. We also detected that some LR HPV types (54, 81 and 83) were found only in HIV + women. The prevalence of multiple HPV infections was 32.5% (13/40) for the HIV + women *versus* 20% (119 / 992) in HIV-negative women. Multiple HPV infections in HIV-positive women included the following types:16, 18, 31, 39, 51, 52, 58, 59, and 68; in one case, infection involved the following types:53, 59, 61, 68 and 84.

## Discussion

This is a pilot study assessing for the first time in Romania the HPV types distribution in HIV-positive women, and their risk for developing precancerous lesions and cervical cancer, was compared to that of HIV-negative women. The HPV type distribution in HIV-negative women in the present study does not bring any new significant information than those previous published [10]:the same high prevalence for HPV types 16, 53, 51, 52, 18 and 31 was observed in a study group that was twice as large (992 versus 514 women genotyped).Therefore, we will focus in this discussion on the results of HPV genotyping in HIV-positive women.

**Fig 1:**
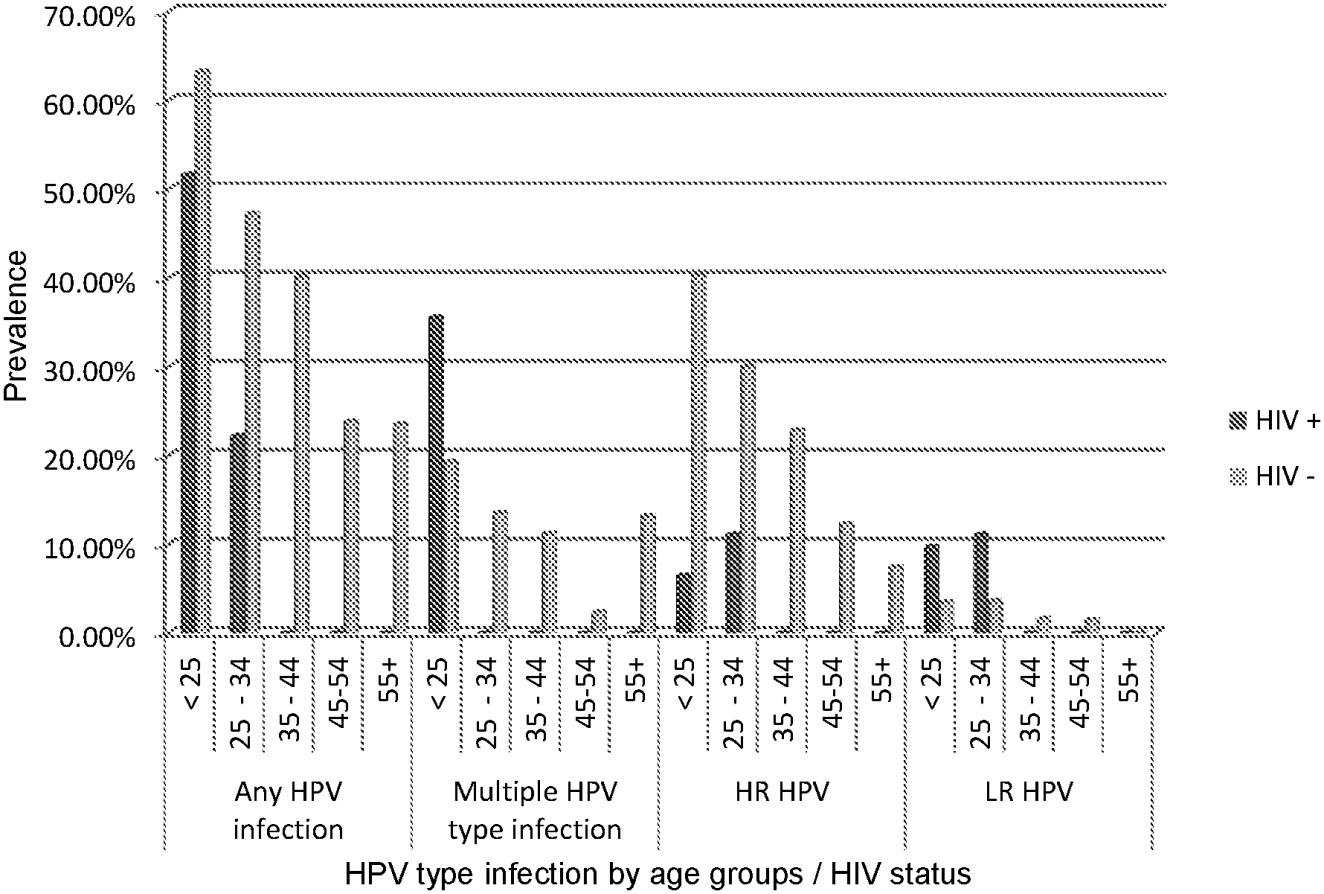
The HPV prevalence in HIV-positive women and HIV-negative women, compared by age group.

In Fig 1 we compared theHPV prevalence in HIV-positive women and HIV-negative women by age group and can be observed that the HPV prevalence in HIV-positive women is higher in the age group < 25 years old, for multiple HPV types infections (OR = 2.30 for multiple HPV type infections). Higher HPV genotype prevalence found for the HIV-positive, can be directly linked to generation-related differences in sexual behavior. In HIV-negative women, the HPV prevalence between women of different generations decreased with age increasing.

We acknowledge that the group of HIV+ women who were infected iatrogenically is very small mainly because in Romania all HIV positive patients are treated and monitorized in 8 regional centers; in Iaşi Center from “Sf Parascheva” Hospital there are around 1000 patients treated with HAART therapy since 2010; therefore, many differences compared to HIV-uninfected women will not be statistically significant even if they are rather strong. The demographic data regarding the tested women are shown in Table 1. We evaluated if HIV represent a risk factor in acquiring HPV infection and we have detected significant association for multiple HPV type infections (OR = 2.30) and LR HPV types (OR = 2.95). After age adjustment of the two study groups, we found like risk factor age < 25 years old, (OR = 5.83), number of birth (OR = 2.94 for 1 and 3.90 for 2), abortion (OR = 2.34 for 2 and 27.29 for three), pregnancy (OR = 17.62 for the group < 25 years old and 47.25 for 25–34), inflammatory Pap test (OR = 2.38). Also, after age adjustment, the overall HPV prevalence was 51.6% in HIV+ versus 63.2% in HIV- women aged under 25, and 22.2% in HPV+ versus 47.2% in HIV- women aged 25–34. We conclude that HPV prevalence is lower in HIV+ than in HIV- women when adjusted for age.

**Table 1:** The demographic characteristics of the two study groups.

CHARACTERISTIC			HIV POSITIVE (n = 40)	HIV NEGATIVE (n = 307) ≤30 YEARS	OR	95% CI	P values for chi-2 test
**AGE**							
	< 25		31 (77,5%)	114 (37,1%)	5.83	2.55÷13.71	0.001
	25–34		9 (22,5%)	193 (62,9%)	0.17	0.07÷0.39	0.001
**BIRTH**							
	< 25						
		1	5 (16,1%)	7 (6,1%)	2.94	0.74÷11.49	0.155
		2	0 (0,0%)	0 (0,0%)	-	-	-
		3	0 (0,0%)	0 (0,0%)	-	-	-
	25–34						
		1	1 (11,1%)	36 (18,7%)	0.55	0.02÷4.53	0.896
		2	1 (11,1%)	6 (3,1%)	3.90	0.48÷26.64	0.726
		3	1 (11,1%)	10 (5,2%)	2.29	0.31÷14.98	0.988
**ABORTION**							
	< 25						
		1	3 (9,7%)	15 (13,2%)	0.71	0.15÷2.88	0.831
		2	3 (9,7%)	5 (4,4%)	2.34	0.41÷12.21	0.484
		3	1 (3,2%)	0 (0,0%)	-	-	0.483
	25–34						
		1	3 (33,3%)	34 (17,6%)	2.34	0.44÷11.28	0.453
		2	0 (0,0%)	9 (4,7%)	0.00	0.00÷14.34	0.870
		3	2 (22,2%)	2 (1,0%)	27.29	2.29÷337.9	0.001
**PREGNANCY**							
	< 25		10 (32.3%)	3 (2,6%)	17.62	3.98÷89.21	0.001
	25–34		3 (33.3%)	2 (1,0%)	47.25	5.08÷531.9	0.001
**PAP TEST**							
**NoPAPtest**							
	< 25		5 (16,1%)	28 (24,6%)	0.59	0.18÷1.83	0.452
	25–34		2 (22,2%)	23 (11,9%)	2.11	0.28÷12.19	0.068
**NORMAL**							
	< 25		20 (64,5%)	46 (40,4%)	2.69	1.10÷6.67	0.028
	25–34		5 (55,6%)	74 (38,3%)	2.01	0.45÷9.26	0.493
**ASCUS**							
	< 25		1 (3,2%)	6 (5,3%)	0.60	0.03÷5.39	0.997
	25–34		0 (0,0%)	25 (13,0%)	0.00	0.00÷4.26	0.525
**ASC-H**							
	< 25		1 (3,2%)	6 (5,3%)	0.60	0.03÷5.39	0.997
	25–34		0 (0,0%)	25 (13,0%)	0.00	0.00÷4.26	0.525
**LGSIL**							
	< 25		0 (0,0%)	12 (10,5%)	0.00	0.00÷1.51	0.049
	25–34		1 (11,1%)	43 (22,3%)	0.44	0.02÷3.60	0.704
**HGSIL**							
	< 25		0 (0,0%)	4 (3,5%)	0.00	0.00÷5.79	0.660
	25–34		0 (0,0%)	12 (6,2%)	0.00	0.00÷10.19	0.960
**INFLAMMATORY**							
	< 25		4 (12,9%)	9 (7,9%)	1.73	0.41÷6.08	0.609
	25–34		1 (11,1%)	9 (4,7%)	2.38	0.34÷16.83	0.932
**HPV PREVALENCE**							
**Any HPV infection**							
	< 25		16 (51.6%)	72 (63,2%)	0.62	0.26÷1.49	0.337
	25–34		2 (22.2%)	91 (47,2%)	0.32	0.04÷1.74	0.261
**Multiple HPV type infection**							
	< 25		11 (35,5%)	22 (19,3%)	2.30	0.88÷5.97	0.096
	25–34		0 (0,0%)	26 (13,5%)	0.00	0.00÷4.07	0.503
**HR HPV**							
	< 25		2 (6,5%)	46 (40,3%)	0.10	0.02÷0.47	0.001
	25–34		1 (11,1%)	58 (30%)	0.29	0.01÷2.38	0.397
**LR HPV**							
	< 25		3 (9,7%)	4 (3,5%)	2.95	0.49÷16.88	0.344
	25–34		1 (11,1%)	7 (3,6%)	3.32	0.42÷22.32	0.802
**CD4/μl**							
			369 (14–774)				
**ARN/HIV copies / mL**							
			8575 (0–343450)				

HIV-positive women are more susceptible to HPV infection associated with multiple types and with loss of detection [11, 12]. These HIV-positive women belong to a special cohort of children who were HIV infected iatrogenically at birth or in the first years of life; the detected prevalence of HPV infection (45%) in HIV-positive women agrees with the results of other studies from other countries that reported HPV prevalence rates ranging from 36.5% to 72.2% [13–18].

The high HPV prevalence in this study group HIV-positive women can be explained by at least two main factors: the level of immunity, as demonstrated by a low medium level of CD4 cells (onaverage369 cells/mm^3^), and by a relatively high ARN/HIV median value (8575 copies⁄mL). For the patients with CD4 > 200 cells/mm^3^ and ARN/HIV < 20,000, the HPV prevalence was 33.33%, while for the patients having CD4 < 200 and ARN/HIV > 20,000,the HPV prevalence was doubled to 66.66%.A similar tendency was detected by Dames DN et al (in 2014), who reported that HR HPV infection increased 7 times in the case of CD4+ ≤ 200 cells/mm^3^ *versus*> 200 in a study group of 167 non-pregnant HIV-positive women [17].

**Fig 2:**
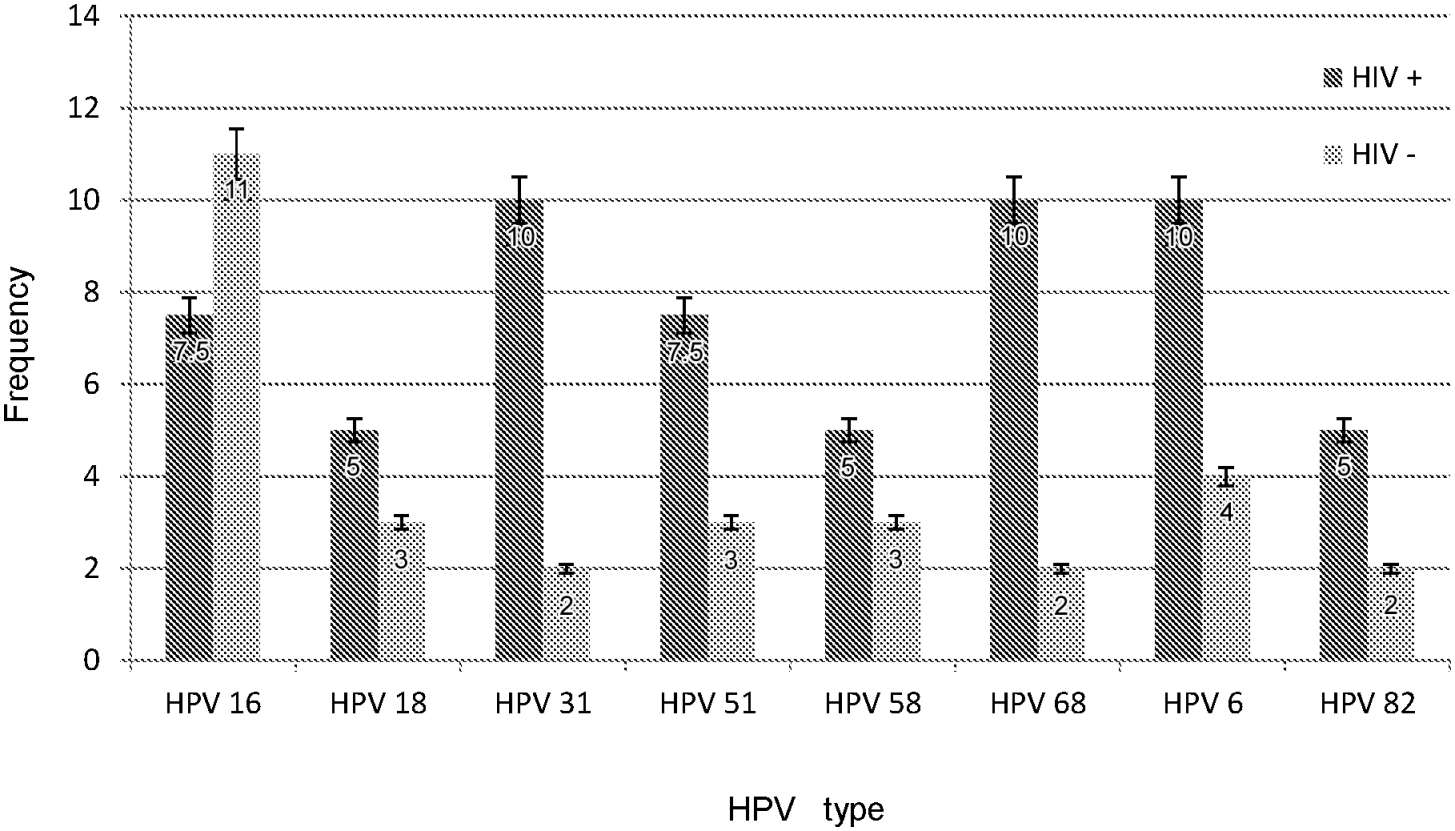
The eight most common HPV types (high-risk or low-risk) for women below age 30, HIV+ /-.

As HPV type distribution is affected by age, we compared in Fig 2 the HPV types distribution to women below age 30 and to the eight most common HPV types (regardless of whether they are high-risk or low-risk), and we observed that the most frequent HPV types, both for HIV+/- women included the 3 types contained in the actually vaccine formula (HPV 16, 18, 6). HPV 16 seems to be relatively under-represented in HIV-positive women, as expected. We are confident in our HPV genotyping results as we have obtained proficiency in HPV genotyping with the LA method in WHO 2010 HPV proficiency testing; even this is a time consuming method, LA does not have the risk of cross-hybridization that can appear in other methods that are used for HPV genotyping, such as enzyme immunoassay [18].The Linear Array HPV Genotyping Strip contains a cross reactive probe (probe line 14) that hybridizes with HPV genotypes 33, 35, 52 and 58. HPV 52 can be interpreted correctly according with the manufacturer’s instructions. We confirmed the HPV 52 type presence with real-time PCR by testing for the E6 oncogene of HPV 52.

One weakness of our study is that 13 of the pregnant HIV-positive women (32.5%) refused second sampling for the cytological examination because these women had a bloody cervix. We respected the patient’s decision in accordance with their informed consent; this possibility could have been avoided if LBC (liquid-based cytology) were available. Regarding the Pap smear examination, only two smears were detected with cytological abnormalities: one had LSIL (low-grade squamous intraepithelial lesion) and infection with HPV 55 and 73, and one had ASC-US (atypical squamous cells of undetermined significance)and AGC (atypical glandular cells) with HPV 31, 61, and 84; the rest of the tested HIV-positive women had a negative Pap smear for intraepithelial lesions or malignity or an inflammatory result despite the high frequency of HPV type infection. Other studies reported an increased prevalence of HPV in association with high-grade lesions in HIV-positive women [12, 13]; in our study, the low number of high-grade lesions can be explained by the median age of the study group (22.9 years), requiring further testing to be able to conclude that the HPV infection was transitory or incident infection instead of persistent. Self-reported numbers of current STD symptoms (4/40) were not connected with any HPV infection.

To assess the risk factors of HIV-positive women for acquiring HPV infections, we analyzed the CD4/μL (< 200, 200–499, > 500), ARN/HIV copies/mL (< 10,000, 10,000–1,000,000, >100,000), age group, number of sexual partners and smoking in relation to the type of HPV infection (single *versus* multiple infections). The Kruskal Wallis score was significant for the number of sexual partners (> 3) and smoking condition (Table 2: The risk factors of HIV-positive women for acquiring HPV infections). For the HIV- negative women, the risk factor for multiple infections was only the number of sexual partners.

**Table 2:** The risk factors of HIV-positive women for acquiring HPV infections.

		HPV TYPE INFECTION			
		Negative	Multiple infections	Single HPV type infection	P value Kruskal-Wallis test
**CD4/μL**					
	< 200	3 (50%)	3 (50%)	0	0.625 ^a)^
	200–499	13 (56.5%)	8 (34.8%)	2 (8.7%)	0.323 ^b)^
	≥ 500	6 (54.5%)	2 (18.2%)	3 (27.3%)	0.959 *)
**ARN/HIV copies⁄mL**					
	< 10,000	12 (60%)	5 (25%)	3 (15%)	0.234 ^a)^
	10,000–100,000	9 (56.2%)	5 (31.3%)	0	0.321 ^b)^
	>10,000	1 (25%)	3 (75%)	0	0.359 *)
**Sexual partners**					
	< 3	21 (75%)	4 (14.3%)	3 (10.7%)	0.001 ^a)^ 0.025 ^b)^
	≥ 3	1 (8.3%)	9 (75%)	2 (.7%)	0.001 *)
**Smoking**					
	No	19 (65.5%)	6 (20.7%)	4 (13.8%)	0.031 ^a)^ 0.737 ^b)^
	Yes	3 (27.3%)	7 (63.6%)	1 (9.1%)	0.034 *)

^a)^ Multiple *vs* negative infections

^b)^ Single HPV type *vs* negative infections

^*)^ HPV positive *vs* negative infections

With the exception of one single case (who refused the therapy), all of the HIV + cases received HAART therapy, which was given for the last 5 years. Antiretroviral therapy was associated with lower prevalence of HPV infections, similar to other authors [14]. Recent studies have reported that vaccines against cervical cancer are efficient even in sexually active women HIV positive; our data regarding the HPV type distribution in HIV + women, even without cervical intraepithelial lesion, supports this vaccine implementation in our country [19].

The most important strength of our study is the characteristics of HIV-positive women who were infected at birth or soon after, and this is the first study, to our knowledge, to assess the HPV type distribution in this category of patients from Romania. Additionally, the genotyping method has good strength and was demonstrated to be proficient by a WHO panel. The weaknesses of this study include the low number of HIV-positive women, explained by the real number of HIV—positive registered in our geographic area, and the lack of biopsies for them, due to the low medium age (22.9 years), which did not allow the gynecologist to see visible lesions in the colposcopic examination. Additionally, the cross-sectional feature of our pilot study did not enabled for investigating the effects of the CD4 count and HAART on HPV infection longitudinally. To study the natural history of HPV infection, it would be necessary to perform one longitudinal study to assess the type of HPV infection, transitory *versus* persistent, considering that persistent HR HPV infections is a major risks for cervical cancer development.

It is well known that only a small proportion of women infected with pathogenic HPV will develop a cancer. Therefore detecting such infections will unnecessarily alarm women and may lead to aggressive intervention from physicians, with a decrease in quality of life and fertility. Screening for pathogenic HPVs is costly. In addition, cervical cancer will develop (although rarely) in HPV- women. The method of choice for a national surveillance program could remain the cytological examination of Pap smears, in the countries in which Pap smears is organized and HPV infection prevalence is low. For our country, in which in 2015 aproximatively 40% of family physicians did not registered themselves in the national cervical screening program (data not published), we can conclude that women registered to those doctors will not be not included in the future screening. In countries where cervical screening is well organized (e.g. Netherlands) it was observed that even with a very well organized cytology based cervical screening programm, the incidence and mortality did not decreased in the last 10 years. For this reason Pap test will be replaced with clinically validated test, more sensitive and specific, being also known that a HR HPV negative woman is having low risk for developing precancerous lesions.

## Conclusions

The HAART therapy and young age seems to be protective against developing precancerous cervical lesions in our study group, even the HPV prevalence and the multiple HPV type distribution were higher than those in the HIV-negative study group. The HPV prevalence in HIV-positive women increased (from 33.3% to 66.6%) in parallel with decreasing immunity. In particular, we noticed that serotypes 54, 81 and 83 were isolated only from HIV-positive patients. All women, regardless of their HIV status, should participate in an organized cervical cancer screening using clinical validated methods at affordable prices.

In our region, it is necessary to improve medical knowledge of general population for a better understanding of the impact of HPV vaccination on the future cervical cancer incidence. Additionally, it would be cost efficient to use an HR HPV clinically validated test for the primary screening of cervical cancer.

## Supporting Information

S1 Table The prevalence of HPV genotypes for HIV negative and HIV positive women. (DOCX)
