# Supplementary material for: The Need for Cervical Cancer Control in HIV-Positive and HIV-Negative Women from Romania by Primary Prevention and by Early Detection Using Clinically Validated HPV/DNA Tests
Source: PLoS One. 2015 Jul 17;10(7):e0132271. doi: 10.1371/journal.pone.0132271 (PMC4506070; doi:10.1371/journal.pone.0132271)
Supplement: S1 Table — (DOCX) [file pone.0132271.s001.docx]

**Additional data:**

**The prevalence of HPV genotypes for HIV negative and HIV positive women**

| **HPV TYPE** | **Percent**  **HIV NEGATIVE** | **Percent**  **HIV POSITIVE** |
| --- | --- | --- |
| 6 | 2,33 | 10 |
| 11 | 0,19 | 5 |
| 16 | 11 | 7,5 |
| 18 | 3,9 | 5 |
| 31 | 3,7 | 10 |
| 33 | 2,14 | - |
| 39 | 0,19 | 2,5 |
| 42 | 1,94 | 5 |
| 51 | 3,7 | 7,5 |
| 52 | 4 | 12,5 |
| 53 | 5,44 | 5 |
| 54 | - | 2,5 |
| 55 | 0,58 | 5 |
| 56 | 0,38 | - |
| 58 | 2,75 | 5 |
| 59 | - | 5 |
| 61 | 0,19 | 10 |
| 66 | 1,36 | 5 |
| 68 | 1,65 | 10 |
| 73 | 1,55 | 12,5 |
| 81 | - | 2,5 |
| 82 | 0,77 | 5 |
| 82 | - | 5 |
| 83 | - | 2,5 |
| 84 | 1,16 | 5 |
| 35, 40, IS39 | 0,19 | - |
| CP6108 | 2,52 | 10 |
| 45 | 2,33 | - |
| 62, 70 | 0,97 | - |
| NEGATIVE | 64,8 | 55 |
